# Glucagon receptor activation contributes to the development of kidney injury

**DOI:** 10.1152/ajprenal.00088.2024

**Published:** 2024-09-12

**Authors:** Anna Billeschou Bomholt, Christian Dall Johansen, Katrine Douglas Galsgaard, Emilie Elmelund, Marie Winther-Sørensen, Jens Juul Holst, Nicolai J. Wewer Albrechtsen, Charlotte Mehlin Sørensen

**Affiliations:** ^1^Department of Biomedical Sciences, University of Copenhagen, Copenhagen, Denmark; ^2^Novo Nordisk Foundation Center for Basic Metabolic Research, Faculty of Health and Medical Sciences, University of Copenhagen, Copenhagen, Denmark; ^3^Novo Nordisk Foundation Center for Protein Research, University of Copenhagen, Copenhagen, Denmark; ^4^Department of Clinical Biochemistry, Copenhagen University Hospital-Bispebjerg Hospital, Copenhagen, Denmark

**Keywords:** albuminuria, diabetic nephropathy, hyperglucagonemia, mesangial cells, podocytes

## Abstract

The underlying causes of diabetic kidney disease are still largely unknown. New insights into the contributing causes of diabetic nephropathy are important to prevent this complication. Hyperglycemia and hypertension are some of the risk factors for diabetic nephropathy. However, the incidence of diabetic nephropathy is increasing despite efforts to normalize blood glucose levels and blood pressure. Therefore, other factors should be investigated as causes of diabetic nephropathy. We investigated whether long-term increased plasma levels of glucagon contribute to the development of pathophysiological changes in kidney function as seen in patients with diabetic nephropathy. Using mouse models of chronic activation and inactivation of glucagon receptor signaling, we investigated whether glucagon is involved in changes in renal function, renal structure, and transcriptional changes. We found several histopathological changes in the kidney, such as thickening of the parietal layer of Bowman’s capsule, glomerular mesangial cell expansion, and significant albuminuria in the mice with activated glucagon receptor signaling. Opposite effects on mesangial area expansion and the development of albuminuria were demonstrated in mice with glucagon receptor inactivation. RNA sequencing data revealed that transcription of genes related to fatty acid metabolism, podocytes, Na^+^-K^+^-ATPase, and sodium/glucose transport was significantly changed in mice with activated glucagon receptor signaling. These data implicate that glucagon receptor signaling is involved in the development of kidney injury, as seen in type 2 diabetes, and that glucagon receptor is a potential therapeutic target in the treatment of diabetes.

**NEW & NOTEWORTHY** This study suggests that the glucagon receptor is a potential therapeutic target in the treatment of diabetic kidney disease. We show, in mice, that long-term treatment with a glucagon analog showed not only pathophysiological changes and changes in renal function but also transcriptional changes in the kidneys, whereas opposite effects were demonstrated in mice with glucagon receptor inactivation. Therefore, the use of glucagon in a treatment regimen requires investigation of possible metabolic and renal abnormalities.

## Graphical abstract

**Figure:**
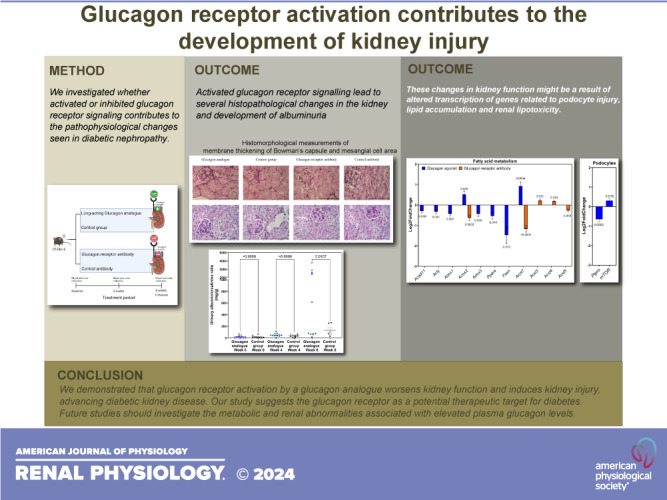

## INTRODUCTION

Diabetic nephropathy affects ∼40% of patients with diabetes and is currently the leading cause of kidney failure (1, 2). Diabetic nephropathy is characterized by significant structural changes in the kidneys, particularly the expansion of the mesangial matrix and the thickening of the glomerular basement membrane (3). Furthermore, urinary excretion of albumin is closely linked to the development of diabetic kidney disease (4, 5) and correlates with increased glomerular extracellular matrix in patients with both type 1 and type 2 diabetes (6, 7). Hyperglycemia (8, 9) and systemic hypertension (10) are some of the major risk factors for complications in patients with diabetes, including nephropathy. However, the incidence of diabetic nephropathy has been gradually increasing despite increasing efforts to normalize blood glucose levels and blood pressure. Therefore, other factors associated with type 2 diabetes should be investigated as the underlying cause of diabetic nephropathy. An important characteristic of type 2 diabetes is increased plasma glucagon concentrations (11). Increased plasma levels of glucagon should normally be suppressed by elevated glucose levels; however, patients with type 2 diabetes have increased plasma glucagon levels even at elevated glucose levels (12) and impaired initial suppression of plasma glucagon concentration after oral glucose ingestion (13). Previously, glucagon infusion has been shown to acutely increase glomerular filtration rate, renal blood flow, and renal excretion of electrolytes in dogs and sheep (14–17), in healthy human subjects (18), and in patients with type 1 diabetes (19). Increased urinary excretion of β_2_-microglobulin, a sign of renal damage, was demonstrated in healthy men when glucagon plasma concentrations were increased by infusion, corresponding to pathophysiological levels seen in patients with dysregulated type 1 diabetes mellitus (18). Furthermore, patients with type 2 diabetes with end-stage renal disease have elevated fasting plasma levels of glucagon, increasing linearly from chronic kidney disease stages 1 to 5 (20). Collectively, the aforementioned findings suggest that glucagon might be involved in the development of pathophysiological changes in renal function seen in patients with diabetic nephropathy. Here, we investigated whether long-term increased plasma levels of glucagon contribute to the pathophysiological changes seen in diabetic nephropathy, using mouse models of long-term activation and inactivation of glucagon receptor signaling.

## MATERIAL AND METHODS

### Animals

This study was performed with permission from the Danish Animal Experiments Inspectorate (License No. 2018-15-0201-01397) and in accordance with EU Directive 2010/63/EU and guidelines of Danish legislation governing animal experimentation (1987) and the United States National Institutes of Health (Publication No. 85-23). The local ethical committee at the University of Copenhagen (P21-337) approved the experiments. C57BL/6JRj mice were obtained from Janvier Laboratories (Saint-Berthevin Cedex, France). Animals were housed in ventilated cages in a standard 12:12-h light-dark cycle with free access to water and standard chow. Only female mice were used in this study, as they exhibit more stable behavior despite having an estrous cycle. Female mice are less aggressive than male mice and up to eight female mice can be housed together. As mice are herd animals and very social, they appreciate being in a group.

### Experimental Setup

**Figure 1.**
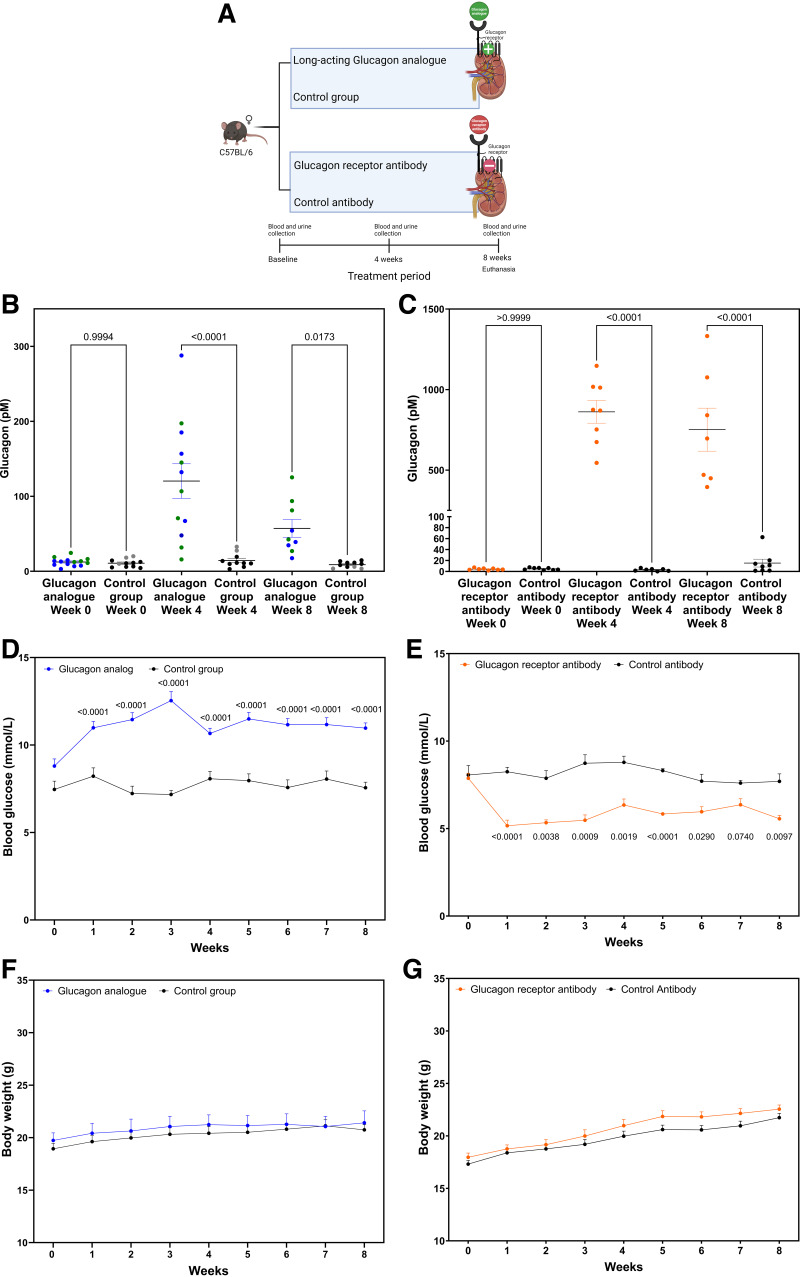
*A*: illustration of the experimental setup, with treatment, and the timeline for the collection time of blood and urine samples. [Created with BioRender.com.] *B* and *C*: plasma glucagon levels in mice treated with the glucagon analog (*B*) and the glucagon receptor antibody (*C*) increased significantly after 4 and 8 wk compared with control mice. Blue and black dots represent C57BL/6 mice, whereas green and gray dots represent GLP-1R KO mice. *D* and *E*: blood glucose levels increased significantly in the analog-treated group (*D*) and decreased significantly in the antibody-treated group (*E*) compared with control mice. *F* and *G*: the body weight increase did not differ from the control groups in either the analog-treated group (*F*) or the antibody-treated group (*G*) when assessed weekly for 8 wk. Data are shown as means ± SE; *n* = 7–14. C57BL/6 mice were 7 wk old at the start of the experiment. GLP-1R KO mice were 9–20 wk old at the start of the experiment. Results in *B* and *C* were assessed by one-way ANOVA and *D*–*G* by two-way ANOVA. GLP-1R KO, Glucagon-like peptide-1 receptor knockout.

Thirty-two female C57Bl/6JRj mice (7 wk of age) received either a long-acting glucagon analog (NNC9204-0043, Novo Nordisk, Bagsværd, Denmark; terminal half-life of 5 to 6 h, 1.5 nmol/kg twice daily), PBS + 1% BSA (twice daily), a long-acting glucagon receptor blocking antibody [REGN1193; Regeneron, Tarrytown, NY, RRID:AB_2783540; IC_50_: 3.5 pM (21), 10 mg/kg, once weekly], or a control antibody (REGN1943; Regeneron, 10 mg/kg, once weekly) (Fig. 1*A*). Drugs were administered over a period of 8 wk by subcutaneous injection. The initial dose of the glucagon analog of 3 nmol/kg was reduced to 1.5 nmol/kg after 16 days of treatment, due to a weight decrease of >20% in one of the mice (the mouse was culled and excluded from the study). A group of nine female glucagon-like peptide-1 (GLP-1) receptor (GLP-1R) knockout (KO) mice, characterized previously (22), was included to control for potential GLP-1R-mediated effects of glucagon treatment, as glucagon is a weak agonist of GLP-1R (23). Six of these mice received the long-acting glucagon analog or PBS + 1% BSA, twice daily, and three served as controls. The GLP-1R KO mice were 9–20 wk of age at the start of the experiment. Apart from plasma glucose levels (Supplemental Fig. S2*B*) and baseline body weight (Supplemental Fig. S2*C*), data from the GLP-1R KO mice were similar to data from the C57Bl/6JRj mice that received either the long-acting glucagon analog or PBS + 1% BSA, and, therefore, the data were pooled, except for RNA sequencing data (Supplemental Figs. S1–S4 provide data comparing GLP-1R KO and C57BL/6 mice for both treated and untreated groups). Once weekly, before injections, the mice were weighed and blood glucose was measured with a handheld glucometer (Accu-Chek Mobile, Cat. No. 05874149001, Roche Diagnostics, Basel, Switzerland). Before the initial injection and after 4 wk, a blood sample (∼150 µL) was collected from the retrobulbar plexus in EDTA-coated capillary tubes (Micro Haematocrit Tubes, Cat. No. 167313, Vitrex Medical, Herlev, Denmark). Furthermore, spot urine was collected from each mouse before the initial injection and after 4 and 8 wk. We experienced inconsistencies in the collection of urine samples, as not all mice urinated during sample collection and the volume of urine was variable. As a result, some analyses, such as urinary urea concentration and the urinary albumin/creatinine ratio, did not include the full number of samples because insufficient urine was collected to measure all parameters. These inconsistencies were primarily due to the method used for collecting urine. To collect the urine, each mouse was fixed in one quick move while being transferred above a weighing dish; in this way, the mouse receives a minor shock and urinates. After 8 wk of treatment, the mice were anesthetized with an intraperitoneal injection of ketamine/xylazine (100/10 mg/kg) diluted in saline solution (0.9% NaCl) and blood was withdrawn from the vena cava to induce exsanguination. The kidneys were removed and weighed; one kidney was snap frozen in liquid nitrogen and stored at −80°C until further analysis, whereas the other was fixed in 10% neutral buffered formalin until preparation for histology.

### Biochemical Analyses

Plasma concentrations of glucagon were measured using a commercial enzyme-linked immunosorbent assay (ELISA) (Cat. No. 10-1281-01, Mercodia, Uppsala, Sweden). Besides endogenous glucagon, this assay also detects the glucagon analog (NNC9024-0043) in plasma but at a weaker reaction. Plasma concentrations of insulin were measured using a commercial ELISA kit (Cat. No. 10-1247-01, Mercodia) (Supplemental Fig. S1). Plasma and urine concentrations of urea were quantified using the QuantiChrom Urea Assay kit (Cat. No. DIUR-100, BioAssay Systems, Hayward, CA). Plasma and urine osmolarity were measured using the K-7400S Semi-Micro Osmometer (KNAUER, Berlin, Germany). The measurement is based on freezing point depression.

To estimate albuminuria, urinary and plasma albumin concentrations were measured using a commercial ELISA kit (Cat. No. E99-134, Bethyl Laboratories, Waltham, MA). The urinary and plasma creatinine concentrations were measured using a modified protocol for creatinine measurement for the cobas 6000 analyzer, using cobas reagents. In short, 15 µL of samples, standards, and controls were incubated in 80 µL R1 creatininase [*N*-tris(hydroxymethyl)methyl-3-propanesulfonic acid, 30 mmol/L, pH 8.1] on an ELISA plate to break down the creatinine. Next, 40 µL of R3 creatininase [*N*-tris(hydroxymethyl)methyl-3-propanesulfonic acid, 50 mmol/L, pH 8.0] was added, and the plate was incubated for 10 min at 37°C. The plate was read as an enzyme kinetic measurement at 700/546 nm.

### Histology

Renal tissue was fixed in 10% neutral formalin buffer (BAF-5000-08A, CellPath, Powys, UK) for 24 h. The tissue was dehydrated and paraffin-embedded. Histological sections of 4 μm were cut and stained with periodic acid-Schiff (PAS) and Jones’ methenamine silver. A total of 42 kidneys were evaluated for glomerular diameter, mesangial expansion, and Bowman’s capsule thickening. The kidneys were cut into longitudinal sections, and for each kidney, five different glomeruli were selected for measurement. Pictures were taken using a light microscope connected to a camera and analyzed in ImageJ (ImageJ, RRID:SCR_003070) or ZEN 3.6 (Blue edition). All measurements were blinded and only glomeruli that had both a urinary pole and a vascular pole were selected for measurement.

For the measurement of glomerular diameter and mesangial expansion, five different glomeruli were selected for measurement for each kidney. The straight line tool was selected in ImageJ to draw a straight line across the glomerulus, thus measuring the diameter. To ensure correct positioning of the glomerulus, both the vascular pole and the urinary pole needed to be present for the glomerulus to be selected for measurement. For measurement of mesangial expansion, images of the glomeruli were converted to 8 bit, and auto threshold was selected. We chose the Shanbhag method, from which there was the best exposure of our pictures, and the contrasts were colored black. The area of mesangial cells was analyzed and quantified. For measurement of Bowman’s capsule, an image was selected in the ZEN 3.6 software and the multiple distances tool was selected under graphics. For each kidney, 5 glomeruli were selected and each of the 5 glomeruli was measured at 10 different locations along the parietal cells lining Bowman’s capsule. This was done by dragging a parallel line to the inner or outer membrane, and 10 measurement lines across that line were drawn to represent the different thicknesses of the membrane.

### RNA Sequencing and Bioinformatic Analysis of RNA Sequencing Data

Kidney tissue was obtained from mice treated for 8 wk with either a glucagon analog, PBS, a glucagon receptor antibody, or a control antibody. The kidney tissue was kept at −80°C until analysis. Total DNA/RNA was purified using an AllPrep DNA/RNA mini kit (Cat. No. 80204, QIAGEN, Hilden, Germany) according to the manufacturer’s instructions and quality tested using a 2100 Bioanalyzer Instrument (Agilent Genomics, Santa Clara, CA). The extracted RNA was treated with DNase using an RNeasy Mini kit (Cat. No. 74106, QIAGEN) and an RNase-Free DNase set (Cat. No. 79254, QIAGEN) according to the manufacturer’s protocol. All the above-mentioned processes were performed on a QIACube machine (QIAGEN). Library preparation was done using TruSeq Stranded Total RNA Library Prep Gold (Cat. No. 20020599, Illumina, San Diego, CA) and IDT for Illumina-TruSeq RNA UD Indexes (Cat. No. 20022371, Illumina) according to the manufacturer’s instructions. RNA sequencing libraries were paired end sequenced (2 × 150 bp) on an Illumina NovaSeq 6000 instrument using an S1 flow cell, and raw sequencing data were processed using bcl2fastq software (v. 2.20.0, Illumina).

The read quality of the raw sequencing data (FASTQ) was evaluated using FastQC (v. 0.11.9). Reads were mapped to a decoy-aware transcriptome (M30/GRCm39) using Salmon (v. 1.9.0) and selective alignment. The data were normalized using the algorithm variance stabilizing transformation offered by the DESeq2 package (24) (v. 1.36.0) in R (v. 4.2.0). The same R package was used to identify differentially expressed genes (false discovery rate < 0.05). Gene Ontology (GO) (25) analysis was performed at the level of biological processes (BPs), with the list of all identified genes (excluding pseudogenes and lowly expressed genes) used as background (24, 26).

### Statistical Analysis of Data (With the Exception of RNA Sequencing Data)

Statistical analyses were performed with GraphPad Prism (GraphPad Software, La Jolla, CA; RRID:SCR_002798). Data are reported as means ± SE. Statistical significance was accepted at values of *P* ≤ 0.05 and assessed by one-way ANOVA, two-way ANOVA, or unpaired *t* tests as specified in the figure and table legends.

## RESULTS

### Pathophysiological Changes Induced by Glucagon

To study the long-term effects of glucagon, we administered a long-acting glucagon analog twice daily or a glucagon receptor blocking antibody once weekly for 8 wk (Fig. 1*A*). We measured plasma levels of glucagon and changes in blood glucose levels to validate the effect of the treatments. The plasma glucagon levels in the mice treated with the glucagon analog were significantly elevated (∼10-fold at 4 wk and 4- to 5-fold at 8 wk compared with the control groups) (Fig. 1*B*). Supplemental Fig. S2*A* provides a comparison between glucagon analog-treated and untreated GLP-1R KO mice and C57BL/6 mice, revealing no significant difference between the two strains. As expected, endogenous glucagon was greatly increased when the glucagon receptor was blocked using the receptor antibody (Fig. 1*C*). Blood glucose levels were significantly elevated throughout the experiment in the analog-treated group and decreased significantly in the glucagon receptor antibody-treated mice, whereas no changes were found in the control groups (Fig. 1*D*). Baseline glucose levels tended to be higher (statistically significant higher from *week 3* to *week 8*) in the GLP-1R KO control mice compared with the wild-type C57BL/6 control mice. GLP-1R KO mice do not exhibit increased insulin secretion, which often results in elevated blood glucose levels (27) (Supplemental Fig. S2*B*). No changes in body weight gain were observed during the course of the experiment when compared with the respective control groups (Fig. 1*E*). The glucagon analog-treated GLP-1R KO mice were older at the start of the experiment, which contributed to their higher overall body weight compared with the C57BL/6 mice throughout the experiment (Supplemental Fig. S2*C*)

### Changes in Renal Function

**Figure 2.**
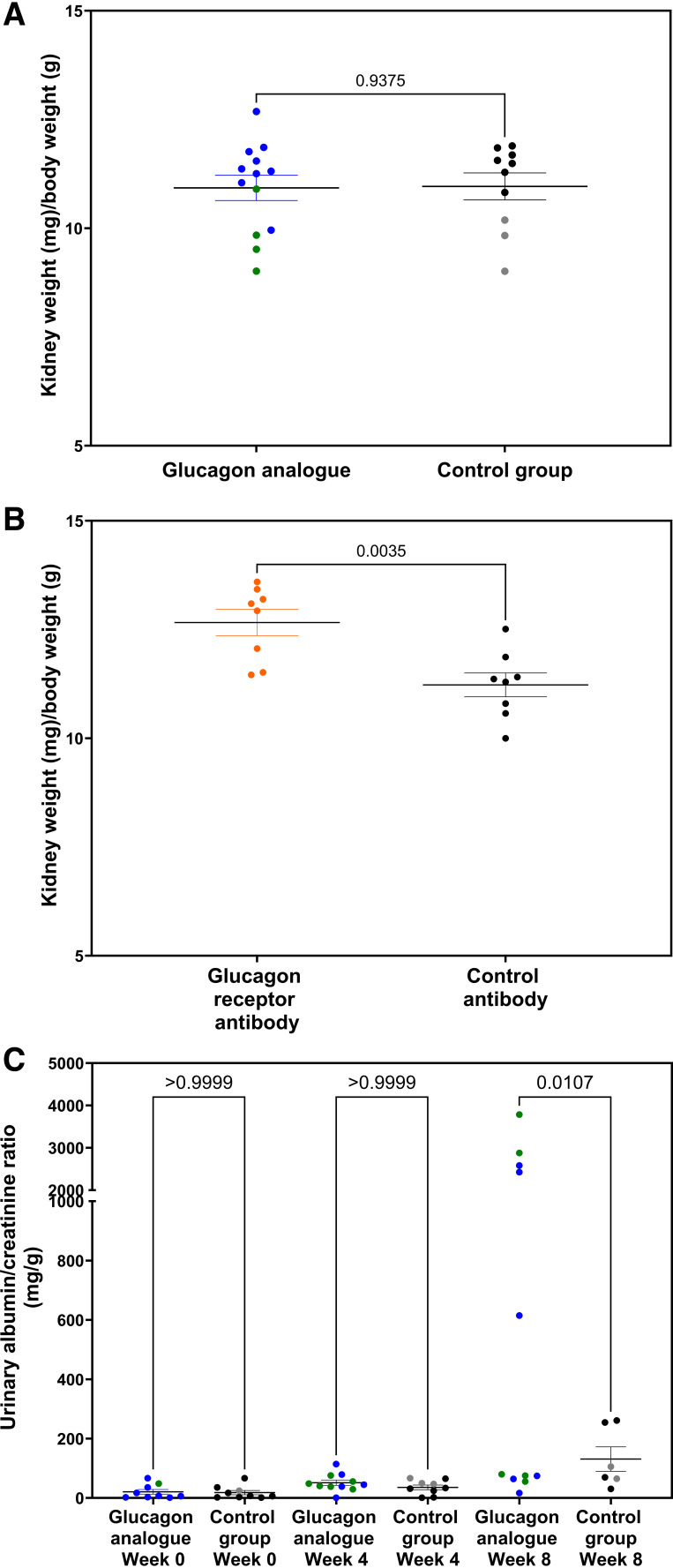
*A*: kidney/body weight ratio did not change after 8 wk of glucagon analog treatment. Blue and black dots represent C57BL/6 mice, whereas green and gray dots represent GLP-1R KO mice. *B*: kidney/body weight ratio increased significantly after 8 wk of glucagon receptor antibody treatment. *C*: urinary albumin-to-creatinine ratio increased significantly after 8 wk of glucagon analog treatment. Blue and black dots represent C57BL/6 mice, whereas green and gray dots represent GLP-1R KO mice. Data are shown as means ± SE; *n* = 8–11. C57BL/6 mice were 7 wk old at the start of the experiment. GLP-1R KO mice were 9–20 wk old at the start of the experiment. Results in *A* and *B* were assessed by unpaired *t* test and *C* by one-way ANOVA. GLP-1R KO, glucagon-like peptide-1 receptor knockout.

To assess the effects of increased glucagon receptor activation on renal function, we measured the kidney weight/body weight ratio and found no difference compared with the control group (Fig. 2*A*). When comparing GLP-1R KO mice with C57BL/6 mice, the GLP-1R KO mice had a significantly lower kidney weight/body weight ratio in both the glucagon analog-treated group and the control group (Supplemental Fig. S3*A*). Furthermore, the glucagon receptor antibody-treated mice had a significant increase in kidney weight/body weight ratio (Fig. 2*B*). When measuring the urinary albumin-to-creatinine ratio, albuminuria was significant after 8 wk of glucagon analog treatment (Fig. 2*C* and Supplemental Fig. S3*B*). Interestingly, only half the mice responded, whereas the other half showed no increase in albuminuria regardless of the mouse strain (GLP-1R KO or C57BL/6). No change in albuminuria was observed in the glucagon receptor antibody-treated mice (Table 2). Furthermore, we found that neither the glucagon analog-treated mice nor the glucagon receptor antibody-treated mice showed changes in plasma urea concentrations (Tables 1 and 2 and Supplemental Fig. S3*C*) or urinary urea concentrations measured in spot urine (Tables 1 and 2 and Supplemental Fig. S3*D*) after 4 or 8 wk of treatment {Supplemental Fig. S3, *C* and *D,* provides a comparison between GLP-1R KO mice and C57BL/6 mice in both treated and untreated groups [nonsignificant (NS)]}. However, the urinary urea-to-creatinine ratio was significantly increased after 4 wk of glucagon analog treatment (Table 1), primarily due to a single GLP-1R KO subject with an unusually high data point.

**Table 1.** Effect of long-term glucagon receptor activation on urea and glomerular diameter

	Baseline Glucagon Analog	Baseline Control Group	*Week 4* Glucagon Analog	*Week 4* Control Group	*Week 8* Glucagon Analog	*Week 8* Control Group
Plasma urea, mg/dL	55 ± 22	53 ± 24	47 ± 22	51 ± 19	61 ± 144	61 ± 19
Urine urea, mg/dL	3,713 ± 393	6,020 ± 4	3,753 ± 1,027	5,117 ± 743	2,788 ± 314	4,046 ± 651
Urinary urea-to-creatinine ratio, mg/dL	267	345 ± 22	704 ± 178*	259 ± 39	269 ± 35	249 ± 75
Glomerular diameter, µm					126.6 ± 1.7	126.0 ± 2.7

Values are means ± SE.

*P ≤ 0.05 compared with the control group by one-way ANOVA.

**Table 2.** Effect of long-term glucagon receptor inhibition on albuminuria, urea, kidney weight, and glomerular diameter

	Baseline Glucagon Receptor Antibody	Baseline Control Antibody	*Week 4* Glucagon Receptor Antibody	*Week 4* Control Antibody	*Week 8* Glucagon Receptor Antibody	*Week 8* Control Antibody
Urinary albuminuria, mg/g	19.8 ± 15.7	15.2 ± 7.5	18.9 ± 4.4	18.9 ± 6.8	59.7 ± 27.1	69.5 ± 26.5
Urine urea, mg/dL		2,686 ± 335	6,240 ± 501	6,211 ± 529	6,056 ± 769	5,390 ± 802
Plasma urea, mg/dL	47 ± 2	52 ± 4	57 ± 4	56 ± 2	67 ± 4	62 ± 7
Urinary urea-to-creatinine ratio, mg/dL		222 ± 45	147 ± 24	179 ± 26	138 ± 22	212 ± 49
Kidney weight, mg					12.7 ± 0.3*	11.2 ± 0.3
Glomerular diameter, µm					130.5 ± 3.2	128.4 ± 3.5

Values are means ± SE.

*P ≤ 0.05 compared with control antibody by an unpaired *t* test.

### Renal Damage Assessed by Histomorphological Measurements

**Figure 3.**
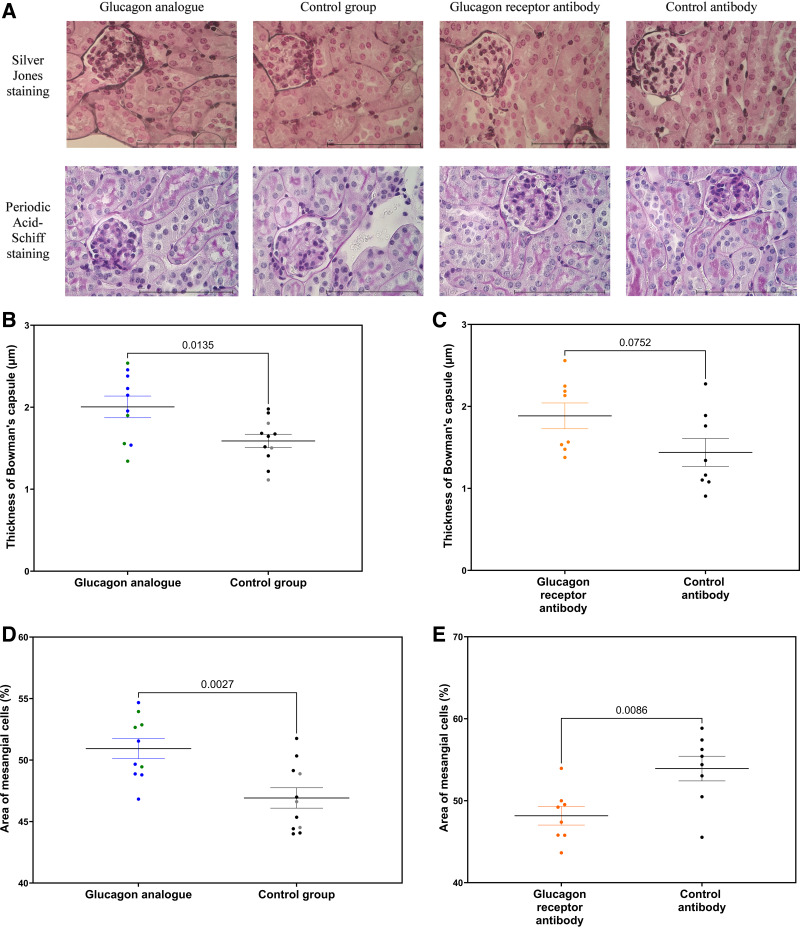
*A*: Jones’ silver staining and periodic acid-Schiff (PAS) staining of a glomerulus in glucagon analog-treated mice and glucagon receptor antibody-treated mice and their respective control mice. *B*: thickness of the parietal layer of Bowman’s capsule in glucagon analog-treated mice, showing a thicker Bowman’s capsule after 8 wk of treatment in the glucagon analog-treated mice compared with their control mice. Blue and black dots represent C57BL/6 mice, whereas green and gray dots represent GLP-1R KO mice. *C*: thickness of the parietal layer of Bowman’s capsule in glucagon receptor antibody-treated mice, showing no difference between the glucagon receptor antibody-treated mice and their control mice. *D*: relative area of mesangial cells in glucagon analog-treated mice, showing mesangial expansion after 8 wk of treatment in the glucagon analog-treated mice compared with their control mice. Blue and black dots represent C57BL/6 mice, whereas green and gray dots represent GLP-1R KO mice. *E*: relative area of mesangial cells in glucagon receptor antibody-treated mice, showing a decrease in mesangial cell area after 8 wk of treatment in the glucagon receptor antibody-treated mice compared with their control mice. Data are shown as means ± SE; *n* = 8–11. C57BL/6 mice were 7 wk old at the start of the experiment. GLP-1R KO mice were 9–20 wk old at the start of the experiment. Results in *B*–*E* were assessed by an unpaired *t* test. Magnification: ×40. Scale bar = 100 µm. GLP-1R KO, glucagon-like peptide-1 receptor knockout.

Renal damage was assessed by histomorphological measurements of mesangial cell area and membrane thickening of Bowman’s capsule. Using Jones’ methenamine silver staining, we found significant membrane thickening of Bowman’s capsule in the glucagon analog-treated mice (Fig. 3, *A* and *B*). Supplemental Figure S4*A* provides a comparison of GLP-1R KO mice and C57BL/6 mice in both treated and untreated groups (NS). No difference in the thickness of Bowman’s capsule was observed in mice treated with glucagon receptor antibody (Fig. 3, *A* and *C*). PAS staining revealed significant mesangial matrix expansion in the glucagon analog-treated mice after 8 wk of treatment (Fig. 3, *A* and *D*). Supplemental Figure S4*B* provides the comparison between GLP-1R KO mice and C57BL/6 mice in both treated and untreated groups (NS). A significant decrease in the area of mesangial cells was found in glucagon receptor antibody-treated mice (Fig. 3, *A* and *E*). No changes were observed when measuring glomerular diameter in either the glucagon analog-treated mice [regardless of genotype (Supplemental Fig. S4*C*] or the glucagon receptor antibody-treated mice (Tables 1 and 2).

### Transcriptional Changes Related to Renal Damage

**Figure 4.**
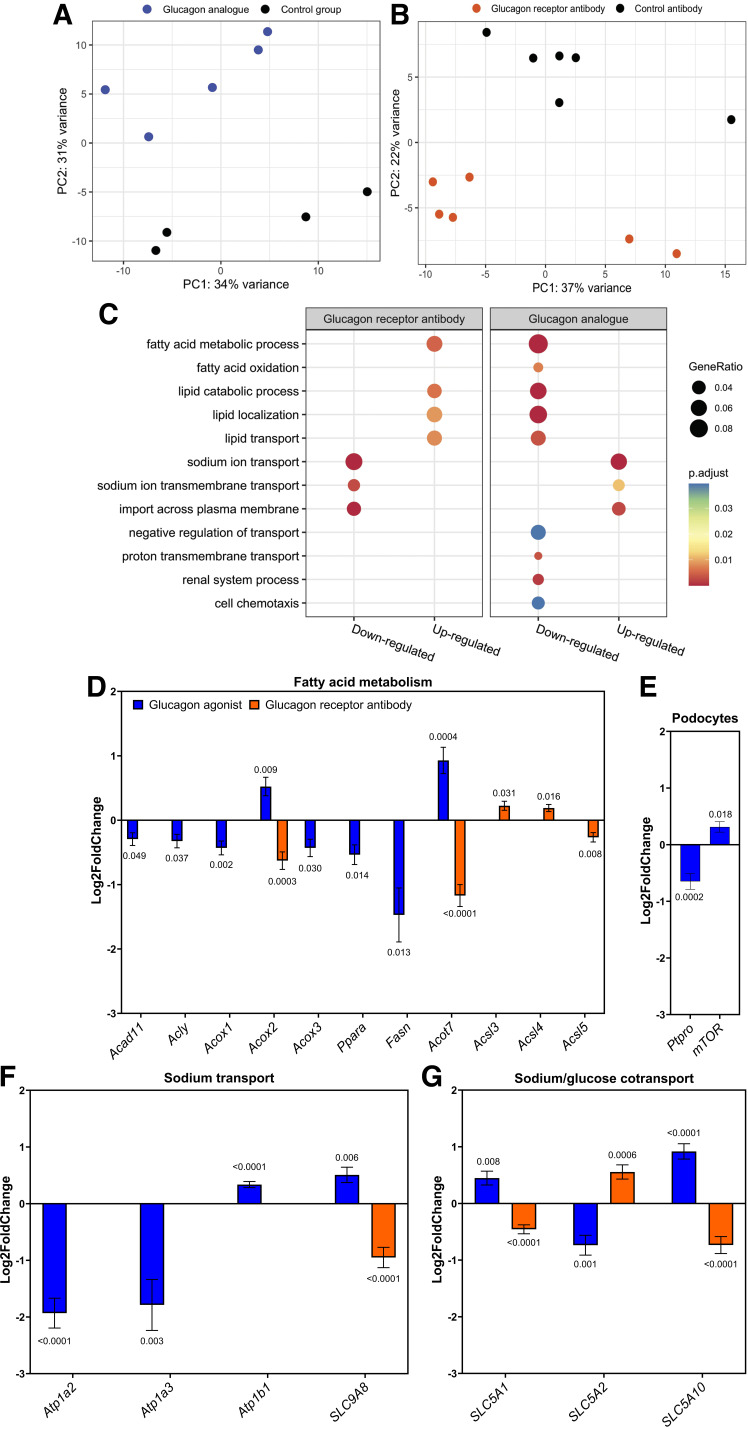
*A*: principal component analysis (PCA) of RNA sequencing samples from mice treated with glucagon analog or PBS showing two separate clusters. *B*: PCA of RNA sequencing samples from mice treated with glucagon receptor antibody or control antibody showing two separate clusters. *C*: Gene Ontology biological processes (GOBP) for up- and downregulated genes in kidney tissue of female mice treated with either a glucagon analog and compared with its control group or a glucagon receptor antibody compared with a control antibody, showing GeneRatio adjusted *P* values. *D*: significant up- or downregulation of transcription of genes associated with fatty acid metabolism (*Acad11*, *Acly*, *Acox1*, *Acox2*, *Acox3*, *Ppara*, *Fasn*, *Acot7*, *Acsl3*, *Acsl4*, and* Acsl5*) in glucagon analog-treated mice compared with their control group or glucagon receptor antibody-treated mice compared with control antibody. *E*: changes in transcription of genes related to cell proliferation regulation of podocytes, with a significant downregulation of *Ptpro* and significant upregulation of *mTOR* in glucagon analog-treated mice compared with their control mice. *F*: changes in genes related to sodium reabsorption, with a significant downregulation of the α_2_ and α_3_ subunits (*Atp1a2* and *Atp1a3*) and an upregulation of the β_1_ subunit (*Atp1b1*) of Na^+^-K^+^-ATPase in glucagon analog-treated mice compared with their control group and transcription of Na^+^/H^+^ exchanger 8 (*SLC9A8*) in glucagon analog-treated mice compared with their control group or glucagon receptor antibody-treated mice compared with control antibody. *G*: significant up- or downregulation of transcription of genes related to sodium/glucose cotransport in glucagon analog-treated mice compared with their control group or glucagon receptor antibody-treated mice compared with control antibody. Data are shown as means ± SE. Results were analyzed by an unpaired *t* test. *n* = 4–6. C57BL/6 mice were 7 wk old at the start of the experiment. *Acad11*, acyl-CoA dehydrogenase; *Acly*, ATP citrate lyase; *Acot7*, acyl-CoA thioesterase; *Acox1*, acyl-CoA oxidase 1; *Acox2*, acyl-CoA oxidase 2; *Acox3*, acyl-CoA oxidase 3; *Acsl3*, acyl-CoA synthetase 3; *Acsl4*, acyl-CoA synthetase 4; *Acsl5*, acyl-CoA synthetase 5; *Fasn*, fatty acid synthase; *mTOR*, target of rapamycin kinase.

To dissect the molecular mechanisms underlying the observed effects on increasing albuminuria and histomorphological changes, we performed RNA sequencing of whole kidney tissue. Global gene expression profiles were evaluated by principal component analysis. The glucagon analog-treated mice clustered together and separated from their control mice (Fig. 4*A*). The same applied for the glucagon receptor antibody-treated mice compared with their control (Fig. 4*B*). Furthermore, an enrichment analysis of GO BPs revealed that genes related to fatty acid oxidation and lipid transport were downregulated in mice treated with glucagon analog and upregulated in mice treated with glucagon receptor antibody (Fig. 4*C*). Notable alterations included changes in the transcription of genes coding for fatty acid metabolism genes and cell proliferation regulators of podocytes. The transcription of the key transcriptional regulator gene *PPARα*, related to fatty acid metabolism, was downregulated in the glucagon analog-treated mice (Fig. 4*D*). A reduction was also found for fatty acid metabolism genes encoding fatty acid synthase (*Fasn*) (Fig. 4*D*), acyl-CoA oxidase 1 and 3 (*Acox1* and *Acox3*) (Fig. 4*D*), acyl-CoA dehydrogenase (*Acad11*) (Fig. 4*D*), and ATP citrate lyase (*Acly*) (Fig. 4*D*), whereas transcription of genes encoding acyl-CoA oxidase 2 (*Acox2*) (Fig. 4*D*) and acyl-CoA thioesterase (*Acot7*) (Fig. 4*D*) was significantly upregulated. In mice with inhibited glucagon receptor activation, the transcription of fatty acid metabolism genes encoding acyl-CoA synthetase 3 and 4 (*Acsl3* and *Acsl4*) was upregulated (Fig. 4*D*), whereas acyl-CoA thioesterase (*Acot7*) (Fig. 4*D*) and acyl-CoA synthetase 5 (*Acsl5*) (Fig. 4*D*) were downregulated. We also searched for genes known to be cell proliferation regulators of podocytes. Here, we found that the protein tyrosine phosphatase receptor type O (*Ptpro*) gene was significantly downregulated (Fig. 4*E*) and that the target of rapamycin kinase (*mTOR)* was upregulated (Fig. 4*E*) in glucagon analog-treated mice.

Our RNA data also revealed that the transcription of genes coding for α subunits in Na^+^-K^+^ ATPase was significantly reduced (*Atp1a2* and *Atp1a3*). Both the α_2_ and α_3_ subunits were greatly downregulated (Fig. 4*F*), whereas transcription of the β_1_ subunit (*Atp1b1*) was upregulated (Fig. 4*F*) in kidneys from mice with increased glucagon receptor activation. Furthermore, transcription of Na^+^/H^+^ exchanger 8 (*SLC9A8*) was upregulated (Fig. 4*F*) in glucagon analog-treated mice, whereas it was downregulated (Fig. 4*F*) in mice with inhibited glucagon receptor activation. Transcription of genes coding for sodium/glucose cotransporters 1 and 10 (*SLC5A1 and SLC5A10*) was significantly upregulated (Fig. 4*G*), whereas transcription of genes coding for sodium/glucose cotransporters 2 (*SLC5A2*) was significantly downregulated (Fig. 4*G*) in glucagon analog-treated mice. In glucagon receptor antibody-treated mice, transcription of genes coding for sodium/glucose cotransporters 1 and 10 (*SLC5A1 and SLC5A10*) was significantly downregulated (Fig. 4*G*), whereas transcription of genes coding for sodium/glucose cotransporter 2 (*SLC5A2*) was significantly upregulated (Fig. 4*G*).

## DISCUSSION

Results from this study demonstrate that increased plasma glucagon levels result in several histopathological changes in the kidney, such as considerable thickening of the parietal layer of Bowman’s capsule and glomerular mesangial cell expansion. The parietal cells lining Bowman’s capsule are mostly important for maintaining the structure in Bowman’s capsule (28). However, the parietal cells are also speculated to undergo reversible changes that affect renal function (29), having the ability to differentiate into podocytes to replace damaged or old podocytes (30–32). The loss or injury of podocytes might have caused the thickening of the parietal layer of Bowman’s capsule within this study, causing the accumulation of abnormal proliferating parietal epithelial cells. In diabetes, podocyte-mediated glomerular basement membrane thickening has been previously shown to also activate parietal epithelial cells to increase extracellular matrix production, causing a thickening of Bowman’s capsule (33). The profibrotic effect of parietal epithelial cells eventually contributes to the formation of sclerotic lesions and mesangial sclerosis (34). Mesangial cell expansion is considered to be caused by the metabolic abnormalities seen in diabetes (35, 36) and was initially believed to be a result of hyperglycemia alone (37) or by an excessive entry of glucose into the mesangial cells through glucose transporters (38). However, recently it was proposed that hyperglycemia is not the direct trigger for mesangial expansion. Studies showed that the upregulation of glucose transport directly into mesangial cells did not replicate the metabolic and renal phenotypes of type 2 diabetes (39, 40). In addition, another study was unable to reproduce the abnormal metabolic and renal phenotypes of type 2 diabetes in mice treated with high glucose intake (41), clearly demonstrating that high glucose alone is not responsible for the mesangial expansion seen under diabetic conditions. Conversely, our results clearly demonstrate an effect of glucagon on mesangial cells, as glucagon-treated mice showed considerable mesangial cell expansion, whereas glucagon receptor antibody-treated mice had significantly decreased mesangial cell area compared with nontreated mice.

Significant albuminuria was observed in this study after 8 wk of glucagon treatment. Albuminuria increases in correlation with the progression of renal damage (42). This has been confirmed by studies demonstrating that progression to end-stage renal disease is reduced when proteinuria is decreased by antihypertensive treatment (43–46). In the present study, urinary albumin excretion was not significantly increased in mice treated with the glucagon receptor antibody, suggesting that the effects are related to enhanced glucagon receptor activation.

The plasma levels of glucagon achieved with the analog in this study are higher compared with the levels seen in patients with both type 1 and type 2 diabetes (47–50). This was necessary to ensure that we could document a potential effect of glucagon receptor activation over a relatively short period of time. Half of all patients with type 2 diabetes and one-third of those with type 1 diabetes will develop kidney disease (2, 51). Previous studies have reported that the mean time to develop diabetic nephropathy after being diagnosed with diabetes can be as long as 16 yr (52, 53). To address the time issue and to achieve the potential negative consequences of chronic active glucagon receptors on the kidneys, we ensured saturated binding of the glucagon receptors in the kidney for 8 wk. This way, we were able to achieve some of the pathophysiological changes seen in diabetic nephropathy.

The twice-daily injections may be partly responsible for the significantly lower increase in body weight in the analog group compared with the antibody group, which were handled weekly. This difference may also reflect an action of glucagon on appetite and food intake (54). However, no changes in kidney weight were observed in the glucagon analog-treated mice compared with control mice.

We observed a significant drop in plasma insulin levels from *week 4* to *week 8* of the glucagon analog treatment. This drop in plasma insulin levels could be attributed to several factors, such as physiological adaptation, where the mice might be adapting to the treatments over time, leading to a reduction in insulin secretion. Chronic exposure to certain compounds can impact the function and health of pancreatic β-cells, potentially leading to reduced insulin production. Furthermore, elevated glucagon levels could influence insulin secretion negatively over time, where sustained high levels might lead to reduced β-cell activity (55). Another factor that could have influenced the insulin levels could be the stress of frequent handling and injections, which might impact the insulin levels (56). Also, treatment with the glucagon analog might affect appetite, food intake, or metabolic rate, which in turn could influence insulin levels. Finally, changes in insulin sensitivity over the course of the experiment might alter insulin secretion. If the mice become more insulin sensitive, less insulin might be needed to maintain glucose.

Another potential limitation of our study is the decision to pool data from the GLP-1R KO mice with the C57BL/6JRj mice in both the treated and untreated glucagon analog group. The data from the GLP-1R KO mice were statistically similar to those from the C57BL/6JRj mice (except body weight and baseline plasma glucose levels), but differences in genetic background and physiology between these groups could introduce minor inconsistencies that are not immediately apparent. Pooling these data allowed us to achieve sufficient statistical power as we had limited amounts of urine samples available, and statistical comparison between the two genotypes did not reveal any biological differences.

Our RNA sequencing data revealed that transcription of the key transcriptional regulator gene *PPARα* was markedly reduced in glucagon analog-treated mice. Furthermore, downregulated transcription of genes encoding enzymes regulating lipid metabolism was also found. Downregulation of *PPARα* activity and expression results in impaired transcriptional regulation of fatty acid oxidation genes, leading to reduced mitochondrial fatty acid oxidation, causing lipid accumulation, renal lipotoxicity, and tubular injury (57–60). Besides dyslipidemia, the downregulation of the *PPARα* gene has also been linked to glomerular matrix expansion, inflammatory cell infiltration, and proteinuria in animal models with diabetic nephropathy (61, 62). Emerging evidence has further indicated that renal lipotoxicity and lipid accumulation cause mesangial expansion and podocyte detachment and death, leading to proteinuria (63–67). Furthermore, glucagon receptor antibody-treated mice within this study had a significant upregulation of transcription of genes encoding Acyl-CoA synthetizes. These are known to be gatekeepers of fatty acid metabolism and play a central role in lipid biosynthesis and fatty acid degradation (68). Thus, the two treatments have opposing effects on genes controlling fatty acid oxidation. The changes observed in the transcription of genes related to the fatty acid metabolic process in this study could indicate that mice treated with the glucagon analog have renal lipotoxicity and lipid accumulation, which might account for the glomerular matrix expansion, excessive albuminuria, and suspected podocyte loss.

Furthermore, transcription of the *Ptpro* gene was decreased in glucagon analog-treated mice. Podocyte injury has been associated with lower expression of *Ptpro* (69, 70). *Ptpro* is a podocyte receptor membrane protein tyrosine phosphatase (71), and this receptor is assumed to play an important role in regulating the structure and function of podocyte foot processes (72). In addition, our RNA sequencing data demonstrated an increased gene activity of *mTOR* in glucagon analog-treated mice. It has been demonstrated that *mTOR* is significantly overexpressed and activated in diabetic nephropathy (73, 74) and that hyperactivated *mTOR* causes podocyte loss (75). The observed changes in *Ptpro* and *mTOR* gene transcription could, therefore, indicate that kidneys from glucagon analog-treated mice have a loss of podocytes and increased podocyte injury. These molecular changes might in part explain the albuminuria observed in this study.

Potential changes in renal electrolyte handling were also assessed. Sodium reabsorption in the kidney is driven by Na^+^-K^+^-ATPase, which maintains a negative membrane potential and low intracellular Na^+^ concentration (76). Our RNA data demonstrated that transcription of the *Atp1a2* and *Atp1a3* genes is greatly downregulated in glucagon analog-treated mice. These genes encode the α_2_ subunit and α_3_ subunit of Na^+^-K^+^-ATPase, which are responsible for the catalytic properties of the enzyme, and the α subunits contain the binding sites for ATP (77). Furthermore, an upregulation of the transcription of the *Atp1b1* gene was observed. The *Atp1b1* gene encodes the β subunit of Na^+^-K^+^-ATPase, which is a smaller glycosylated membrane protein that is required for normal enzymatic activity (78, 79). It has previously been recognized that Na^+^-K^+^-ATPases are involved in the development of renal injury, where decreased Na^+^-K^+^-ATPase activity causes kidney injury in both in vitro cell models (80) and a nonobese diabetic mouse model (81). Affirmatively, it was demonstrated that the monoclonal antibody DRm217, which is a direct activator of Na^+^-K^+^-ATPase, significantly improved renal function and attenuated apoptosis of renal tubular cells and tubulointerstitial injury in 5/6 nephrectomized rats (82). These findings correlate with our data, pointing to impaired function of Na^+^-K^+^-ATPase in glucagon analog-treated mice, which may, therefore, act as a key facilitator of renal disease progression.

Our RNA sequencing data also revealed an increased transcription of the gene encoding sodium/glucose cotransporter 1 (SGLT-1) and a decrease for sodium/glucose cotransporter 2 (SGLT-2) gene transcription in the glucagon analog-treated mice. Conversely, the gene transcription for SGLT-1 was downregulated in the glucagon receptor antibody-treated mice, whereas the SGLT-2 gene transcription was upregulated. SGLT-1 and SGLT-2 are the major glucose cotransporters in proximal tubules (83–85) with 90% of filtered glucose being reabsorbed via SGLT-2 (86, 87). Interestingly, the increased plasma levels of glucose found in patients with diabetes seem to increase the expression of SGLT-2 in the proximal tubules, which is contrary to our data. However, pharmacological inhibition of SGLT-2 in diabetic rat models has been shown to upregulate the expression of SGLT-1 (88). The physiological consequence of the present changes in gene expression is unknown.

In conclusion, the present study demonstrates that long-term glucagon receptor activation may result in the development of kidney injury, similar to type 2 diabetes, including thickening of the parietal layer of Bowman’s capsule, mesangial area expansion, formation of albuminuria, possibly podocyte loss and decreased Na^+^-K^+^-ATPase activity. Conversely, a decrease in mesangial cell area, absence of albuminuria, and increased kidney weight were found when the glucagon receptor was inhibited. Our study suggests that the glucagon receptor is a potential therapeutic target in the treatment of diabetes and dyslipidemia (89). Hence, it is necessary for future studies that aim to use glucagon in a treatment regimen to investigate the metabolic and renal abnormalities that come with increased plasma glucagon levels.

## DATA AVAILABILITY

RNA sequencing results generated are available through the following apps: https://weweralbrechtsenlab.shinyapps.io/glucagonreceptorantibody/ and https://weweralbrechtsenlab.shinyapps.io/glucagonanalogue/. Raw RNA sequencing data are available at https://www.ebi.ac.uk/biostudies/ under Accession Nos. E-MTAB-12520 for the glucagon analog and E-MTAB-12518 for the glucagon receptor antibody.

## SUPPLEMENTAL MATERIAL

10.6084/m9.figshare.26096161 Supplemental Figs. S1–S4: https://doi.org/10.6084/m9.figshare.26096161.

## GRANTS

This work was supported by the Augustinus Foundation (20-1914 and 21-1518) and the Novo Nordisk Fonden (NNF21OC0070308).

## DISCLOSURES

No conflicts of interest, financial or otherwise, are declared by the authors.

## AUTHOR CONTRIBUTIONS

A.B.B., K.D.G., E.E., M.W.-S., J.J.H., N.J.W.A., and C.M.S. conceived and designed research; A.B.B., C.D.J., K.D.G., and E.E. performed experiments; A.B.B., C.D.J., and C.M.S. analyzed data; A.B.B., C.D.J., and C.M.S. interpreted results of experiments; A.B.B. and C.D.J. prepared figures; A.B.B. and C.M.S. drafted manuscript; A.B.B., C.D.J., K.D.G., E.E., M.W.-S., J.J.H., N.J.W.A., and C.M.S. edited and revised manuscript; A.B.B., C.D.J., K.D.G., E.E., M.W.-S., J.J.H., N.J.W.A., and C.M.S. approved final version of manuscript.
